# Genetic Characterization of *Echinococcus granulosus sensu stricto* Isolated from Human Cysts from Sardinia, Italy

**DOI:** 10.3390/diseases11030091

**Published:** 2023-06-27

**Authors:** Cinzia Santucciu, Piero Bonelli, Angela Peruzzu, Alessandro Fancellu, Antonella Farà, Scilla Mastrandrea, Giovanni Drocchi, Antonio Cossu, Stefano Profili, Alberto Porcu, Giovanna Masala

**Affiliations:** 1 WOAH and NRL for Echinococcosis, Animal Health, IZS della Sardegna, 07100 Sassari, Italy; piero.bonelli@izs-sardegna.it (P.B.); angela.peruzzu@izs-sardegna.it (A.P.); giovanna.masala@izs-sardegna.it (G.M.); 2 Department of Medical, Surgical and Experimental Sciences, University of Sassari, 07100 Sassari, Italy; afancel@uniss.it (A.F.); alberto@uniss.it (A.P.); 3 Department of Biomedical Sciences, Institute of Pathology, University of Sassari, 07100 Sassari, Italy; antonellam.fara@tiscali.it (A.F.); cossu@uniss.it (A.C.); 4 Unità Operativa Complessa di Malattie Infettive, Azienda Ospedaliero–Universitaria, 07100 Sassari, Italy; scilla.mastrandrea@aousassari.it; 5 Department of Surgical and Diagnostic Integrated Sciences (DISC), University of Genova, 16132 Genova, Italy; g.drocchi@hotmail.it; 6 Unità Operativa Complessa di Radiologia, Azienda Ospedaliero-Universitaria, 07100 Sassari, Italy; stefano.profili@aouss.it

**Keywords:** cystic echinococcosis, *Echinococcus granulosus sensu lato*, human diagnosis, molecular characterization, phylogenesis

## Abstract

This study involved 20 patients affected by cystic echinococcosis (CE) who were referred to different hospitals of Sardinia (Italy) from 2017 to 2022. By means of a multidisciplinary approach, diagnosis was confirmed for CE in 18 patients and for different aetiologies in two subjects. Moreover, serology was positive for 15 subjects. Since multiple CE cysts were found in five patients, a total of 27 lesions were collected; however, only one for each patient was investigated for genetic characterization of *E. granulosus s.s.* DNA isolates. Our results included 15 fertile cysts that underwent DNA extraction and amplification by three different PCRs targeting nuclear (*calreticulin*) and mitochondrial genes (cox1 and nad5). DNA was sequenced, and by neighbour-joining phylogenetic trees we determined 10 G1 and five G3 genotypes previously reported in Sardinia. These sequences were used to construct a network, along with those circulating in Mediterranean areas. The haplotype network calculated on cox1 evidenced seven different haplotypes of the 15 isolates, with SAR2 the most represented, carried by seven cysts, and SAR17 never described in the Mediterranean area. Meanwhile, the nad5 sequences showed the most common haplotype as nd5SAR7, as well as two new haplotypes not previously described, nd5SAR13, isolated from a Sardinian patient, and nd5SAR14, isolated from a Romanian patient.

## 1. Introduction

Cystic echinococcosis (CE) is a zoonotic disease caused by *Echinococcus granulosus sensu lato* (*s.l.*), the larval form of a tapeworm belonging to the Cestoda class and the Taeniidae family [1,2,3].

*E. granulosus s.l*. is listed second in the global ranking of the most important foodborne parasites, as reported by the United Nations, the World Health Organization, and the Food and Agriculture Organization [4]. In addition, according to the European ranking, *E. granulosus s.l.* is among the top four foodborne parasites due to its high public health relevance and its significant contribution to the disease burden [5]. CE also represents one of the 20 Neglected Tropical Diseases, according to the World Health Organization [6].

The life cycle of *E. granulosus s.l.* involves definitive hosts, dogs or other canids, which harbour the adult form of the parasite; and intermediate hosts, sheep or other ruminants, in which the larval metacestode can develop in the internal organs [1]. Humans are dead end intermediate hosts since there is no subsequent human-to-dog transmission, but they can accidentally acquire the infection by ingesting eggs that release oncospheres able to penetrate the intestine wall. The oncospheres enter into circulatory and lymphatic systems and reach first the liver (from 70% to 85% of cases) and then the lungs (from 15% to 47%), where they can grow, and one or seldom multiple CE cysts may arise. Only the larvae that escape these filters may arrive at other organs, such as the spleen (from 0.9% to 8%), kidney (from 2% to 4%) [7], brain (1%), and pancreas (from 0.14% to 2%) [8,9]. CE cysts might remain asymptomatic for years [10,11,12] or show clinical signs with unpleasant consequences caused by cyst growth and compression of surrounding anatomical structures, such as rupture in some cases [13].

*E. granulosus s.l.* has a global distribution, triggering several problems for public health and economic losses in humans and livestock [14,15,16]. In particular, the burden of infection is largely relevant in endemic areas associated with pastoral communities, related to poor hygienic standards and close contact between humans and dogs [17,18]. In addition, the presence of a large number of dogs and the uncontrolled slaughter of livestock are important risk factors for human and animal infection, and they enable the spread of CE [19].

Evidence has indicated that CE is widespread in south and southeast Europe [19] and endemic to the Mediterranean basin [20]. In particular, in Italy, the CE incidence corresponds to an average of 1.6/10^5^ inhabitants per year. The Italian average incidence rates are related to a cost of more than 53 million euros and about 3000 disability-adjusted life years (DALYs), as shown by a recent report based on hospital discharge records (HDRs) evaluated from 2001 to 2014 [21]. Despite CE being widespread throughout the country, several differences have been reported among the Italian regions, which are classified as sporadic, endemic, or hyperendemic [21]. In particular, the islands and southern regions (e.g., Sardinia, Sicily, Campania, and Basilicata) present the highest prevalence rates in humans and animals, including even among the European countries [19]. Furthermore, the highest annual average of costs and DALYs [21], along with the major average incidence rate of CE, equal to 6.8/10^5^ and 4.0/10^5^, were reported in Sardinia and Sicily, respectively [22].

*E. granulosus s.l.* genetic variability implies differences in infectivity, pathogenicity, biology, host range, antigenicity, developing rate, and morphology in both medical and veterinary fields [2,14,23,24].

Molecular studies of *E. granulosus s.l.* have identified five distinct species: *E. granlosus sensu stricto* (*s.s.*) (genotypes G1 and G3), *E. equinus* (genotype G4), *E. ortleppi* (genotype G5), *E. canadensis* (genotypes G6–G8 and G10), and *E. felidis* [1,23,25,26,27,28,29,30,31,32].

*E. granulosus s.s.* is widely spread all over the world; in fact, it is the aetiologic agent of the majority of the human CE cases (at least 90%) [33] and is highly endemic in Southern Europe [34] and other Mediterranean countries [2,15,20,35,36,37,38,39,40].

Today, the existence has been confirmed of only two genotypes belonging to *E. granulosus s.s.*, G1 and G3, because of the reconsideration of genotype G2 as a microvariant of G3, based on studies of the nearly complete mitochondrial (mt) genome [29,40]. Previous studies of the molecular characterization of *E. granulosus* have been based on the Oxidase subunit I (cox1) mitochondrial gene [32,41,42,43,44]. Only recently has the analysis of larger portions of the mt genome shown that utilization of this gene marker could not provide an efficient genotypic distinction of *E. granulosus s.s.* [40,45]. Recent studies performed on a large portion of *E. granulosus s.l.* mtDNA demonstrated that genotypes G1 and G3 are distinct by 37 mutations [29,40] and that a short fragment of the mt gene Nicotinammide Adenin Dinucleotide 5 (nad5), different from other mitochondrial markers, allows for adequate discrimination between the two genotypes [30].

The main purpose of this study was to perform the genetic characterization of *E. granulosus s.l.* DNA isolates collected from human cysts of patients referred to different hospitals of Sardinia, Italy. In particular, we aimed to determine the species, genotype, and haplotype of these isolates by sequence analysis of cox1 and nad5 mtgenes.

## 2. Materials and Methods

### 2.1. Geographical Area of the Study

Sardinia, one of the 20 regions of Italy, is an island located in the Mediterranean Sea, with an area of 24,100 km^2^. It is the second largest after Sicily. It is situated between 38°51′ and 41°18′ north latitude and 8°8′ and 9°50′ east longitude to the west of the Italian Peninsula, south of the French island Corsica and north of northern Africa.

### 2.2. Human Patients

This study involved 20 patients investigated from 2017 to 2022, who referred to different Sardinian hospital wards (Italy) (Table 1). Eleven subjects were male and nine female, their age ranging between 18 and 80 years old (55.2 ± 18.4). Four patients living in Italy were from different countries: China (hCE6), Romania (hCE13), Ghana (hCE15), and Morocco (hCE18). An echinococcal cyst was detected as an occasional finding only in a minority of the group since most of the subjects presented with clinical symptoms suggestive of CE. Hepatomegaly, pain, nausea, abdominal discomfort, and jaundice were mainly associated with patients with abdominal CE cyst localization; conversely, the patients with lung lesions reported dyspnoea, chest pain, and coughing. Since unwanted complications related to the neoformations were present due to compressive effects on surrounding vital structures, the 20 patients underwent surgical removal of the cysts [46] and were regularly followed up. The entirety of the medical procedures and ultrasound cyst classification in this study was performed according to the WHO-IWGE guidelines [14,47].

### 2.3. Ethical Statement

Investigations of human patients involved in this study and the relative samples, were performed in agreement with the ethical standard regulations of the Declaration of Helsinki of 1975, revised in 2013. In addition, since 26 March 2013, our Reference Laboratory for Echinococcosis in Sardinia (Italy) has received permission (Protocol n° 1136) to manage and analyse human samples from the ethics committee of the Local Health Authority, following a request of the National Health Service Doctors. Informed consent was also acquired from all patients, and all samples were anonymized before the analysis.

### 2.4. Patient Investigations

*Imaging techniques*. Ultrasound (US), classical radiography, and computed tomography (TC) examinations were performed to display localization, stadium, number, and size of the cysts.

*Immunological tests*. Blood serum samples obtained from each patient were screened for detection of antibodies against *E. granulosus* by an “Echinococcus IgG” ELISA kit (DRG Instruments GmbH, Marburg, Germany) with the wells coated with an *E. granulosus* antigen and confirmed by echinococcus western blot IgG (LDBIO-Diagnostics, Lyons, France), using a whole larval antigen from *Echinococcus multilocularis*. The assays were used according to the manufacturers’ instructions [48].

*Surgical procedures*. Severe complications (Table 1) arising in the patients required removal of the lesions (Table 1) for all 20 patients evaluated [46]. Afterwards, biologic materials were delivered to the WOAH/CeNRE Laboratory for Echinococcosis (Sardinia, Italy) for further examinations.

*Parasitological examination*. Cystic lesions were macroscopically analysed in a polypropylene tray (25 × 31 × 6.5 cm), under a laminary flow hood to define morphological characteristics (size, consistency, shape), and their content was examined for the presence of hydatid fluid and daughter cysts. Cystic liquid (if present) was observed under a stereomicroscope (40×) to detect the presence of protoscoleces. CE cysts were classified as “fertile” or “non-fertile” based on the presence or absence of protoscoleces, respectively. Parasite material was sectioned into different fractions and collected for further investigations.

*Histopathological analysis*. An aliquot from each cystic sample, collected during previous examinations, was fixed in 10% neutral buffered formalin and embedded in paraffin following routine laboratory protocols. Sections were cut serially from paraffin blocks 3–4 µm in size and were stained with haematoxylin and eosin. Microscopic observation was performed at 4×, 10×, and 40× to identify typical features of the parasite corresponding to three distinct characteristics: a thick, acellular, laminated layer with characteristic acidophilic staining; cellular germinal layer; and brood capsules or protoscolices. A host produced granulomatous reactions could be seen surrounding the cyst [49].

**Table 1:** Anamnestic data of the patients involved in the study and morphology and topography of the CE cysts following surgical investigations.

Cyst/Patients	Patient’s Data			Surgical Investigations
ID	Gender	Age	Nationality	Morphology and Topography of CE Cysts
hCE1	Female	68	Italian	Large hepatic cyst (6 × 7 cm) adherent to adjacent hepatic veins
hCE2	Male	53	Italian	Giant hepatic cyst (17 × 15 cm) with daughter cysts
hCE3	Female	55	Italian	Multiple (*n* = 2) giant hepatic cysts (total largest diameter 13 × 10 cm)
hCE4	Male	78	Italian	Large hepatic cyst (8 × 9 cm) compressing hepatic veins
hCE5	Male	49	Italian	Recidival giant fertile hepatic cyst (12 × 14 cm) close to the portal vein
hCE6	Male	41	Chinese	Recurrent large hepatic cyst (8 × 7 cm) complicated by suppurative inflammation and septic shock
hCE7	Female	47	Italian	Recidival multiple giant cyst located in the liver (the largest 13 × 7 cm), infiltrating the diaphragm and the chest wall
hCE8	Female	60	Italian	Large hepatic cyst (7 × 8 cm) in contact with the inferior vena cava causing recurrent cholangitis
hCE9	Female	75	Italian	Large hepatic cyst (6 × 7 cm) causing recurrent cholangitis
hCE10	Male	43	Italian	Large hepatic cyst (7 × 10 cm) causing biliary obstruction and blockage of the bile duct system with jaundice and severe cholangitis
hCE11	Male	75	Italian	Multiple (*n* = 3) large hepatic cysts (from 3 to 7 cm) with obstructive jaundice
hCE12	Male	64	Italian	Large hepatic cyst (11 × 11 cm) adherent to the diaphragm with biliary–bronchial fistula
hCE13	Female	42	Romanian	Multiple (*n* = 2) large hepatic cysts (from 7 to 10 cm) with recurrent cholangitis
hCE14	Male	69	Italian	Polylobulated large pancreatic cysts (7 × 5 cm and 2 × 2 cm)
hCE15	Male	32	Ghanaian	Multiple (*n* = 2) large fluid cysts in the lung (1.3 × 9 × 1.2 cm) and in the kidney (1.3 × 1.0 × 1.2 cm), compressing organs [50].
hCE16	Male	20	Italian	Large hepatic cyst (10 × 9 cm) compressing the hepatic veins
hCE17	Male	80	Italian	Large hepatic cyst (6 × 7 cm) with signs of suppurative inflammation
hCE18	Female	18	Moroccan	Multiple (*n* = 3) large hepatic cysts (larger than 6 × 7 cm) adherent to hepatic portal vein
hCE19	Female	67	Italian	Large hepatic cyst (7 × 8 cm) with fistulation causing biliary obstruction with recurrent cholangitis
hCE20	Female	67	Italian	Large hepatic cyst (7 × 6 cm) adherent to the hepatic portal vein

### 2.5. Molecular Analyses

*DNA extraction.* A portion from each cystic sample, collected during parasitological examination, was placed in a Petri dish (9 cm diameter), and when present, hydatid fluid was collected. The germinal layer was separated, washed twice in phosphate-buffered saline (PBS), and gently scraped to isolate protoscoleces. Hydatid fluid and parasite material were then centrifuged for 10 min at 11,000× *g,* and an aliquot of 25 mg of each sample’s pellet was frozen at −80 °C. Total genomic DNA was extracted using the DNeasy Blood and Tissue Kit (Qiagen, Hilden, Germany) and, the concentration and purity were measured by the NanoPhotometer^®^ N120 (Implen GmbH, Munich, Germany) according to the manufacturer’s instructions.

*DNA amplification.* The DNA samples (*n* = 15) were analysed by three different PCRs (Table 2). The protocols already described were PCR *Echinococcus granulosus sensu stricto (E.g.s.s.)* [51] amplifying nuclear (n) gene targets to identify in one step *E. granulosus s.s.;* and PCR cox1 [52] and PCR nad5 [30], targeting two mtgenes for genotyping and phylogenetic study.

*DNA sequencing.* Amplification products of cox1 and nad5 PCRs were purified by a QIAquick PCR Purification Kit (Qiagen, Hilden, Germany) and were sequenced. Sanger sequencing was performed on both strands on an ABI-PRISM 3500 Genetic Analyzer (Applied Biosystems, Foster City, CA, USA) with a BigDye Terminator Cycle Sequencing kit (Applied Biosystems). The aligned DNA sequences were manually edited by BioEdit software, version 7.0.0, and trimmed to total lengths of 720 bp and 670 bp, respectively. The obtained sequences were examined by BLAST and compared with sequences available in the NCBI database and used for phylogenetic reconstruction.

### 2.6. Phylogenetic Analyses

The consensus sequences of cox1 and nad5 partial mtgenes related to the 15 DNA isolated from each cysts (from hCE2 to hCE16) were deposited in GenBank, edited, and aligned using the ClustalW algorithm implemented in BioEdit Software, version 7.0.0 [53].

Their accession numbers are available for the cox1 mtgene sequences and correspond to: MK780827 for hCE2, hCE4, hCE7, hCE13, hCE14, hCE15, and hCE16h; MK780828 for hCE3, hCE11; MK780843 for hCE5, and hCE8; MK780842 for hCE6; MK780830 for hCE9; MK780839 for hCE10; and MT991983 for hCE12. Moreover, the nad5 mtgene sequences corresponded to MT993968 for hCE2, hCE4, hCE7, and hCE14; MT993965 for hCE3 and hCE11; MT993971 for hCE5; MT993973 for hCE6; MT993970 for hCE8, hCE10, and hCE12; MW287329 for hCE9; and MW287330 for hCE13; MT993962 for hCE15 and hCE16.

Two distinct neighbour-joining (NJ) phylogenetic trees, by means of the Kimura 2 parameter algorithm and implemented in MEGA X software version 10.0.5 [54], were built on the cox1 and nad5 partial DNA sequences obtained in this study and the following reference sequences retrieved from GenBank: *E. granulosus s.s.* G1: NC044548; *E. granulosus s.s.* G3: KJ559023; *E. equinus* G4: AF346403; *E. ortleppi* G5: AB235846; *E. canadensis* G6: AB208063; *E. canadensis* G7: AB235847; *E. canadensis* G8: AB235848; *E. canadensis* G10: AB745463; and *Taenia solium*: AY211880.

The evolutionary model that best fit the data was determined by means of JmodelTest software, version 2.1.7 [55], and the evolutionary history was inferred in Mega X [54].

The haplotypes of the cox1 and nad5 mtgene fragments were identified by DNAsp software, version 6.10.04 [56]. Phylogenetic haplotype networks were constructed based on TCS criteria by means of PopART software version 1.7.1 (https://popart.maths.otago.ac.nz/how-to-cite/) [57]. A BLAST analysis of the haplotypes identified in this study, for sequence alignment and homology searches with sequences available in the NCBI database, was also performed. Moreover, the basis of the BLAST analysis for the specific markers was used to identify nucleotide sequences with a query cover of 100%, and a percentage of identity less than 100% was considered a haplotype that was never before described.

## 3. Results

### 3.1. Patient Findings

In Table 1, principal anamnestic information and surgical findings of the 20 human patients involved in this study are summarized. A total of 27 cystic lesions were collected since multiple cysts were found in patients identified as hCE3 (*n* = 2), hCE11 (*n* = 3), hCE13 (*n* = 2), hCE15 (*n* = 2), and hCE18 (*n* = 3) (for patients with multiple lesions, only one cyst was considered in this study). In the majority of cases, cystic formations were found in the liver (*n* = 24–88.8%); only in three patients were cysts located, respectively, in the pancreas (*n* = 1–3.7%), the lung (*n* = 1–3.7%), and the kidney (*n* = 1–3.7%).

*Imaging techniques*. Imaging investigations of CE lesions (Table 3) revealed a wide range of US stadiation (from CE1 to CE5) and size (from 3 cm to 20 cm). The CE cyst with lung localization in patient hCE15 was detected by traditional radiography and confirmed by TC, with the ability to add several details regarding surrounding organs and blood vessels (Table 3).

*Immunological tests*. Antibodies against *E. granulosus* spp. were detected from 15 positive serum samples by ELISA and were confirmed by WB (Table 3).

*Surgical procedures*: Size, number, and localization were confirmed at the time of surgery.

*Parasitological examination*. The parasitological examination of the 20 cystic lesions revealed that 18 presented typical parasitological features attributable to a CE cyst, and their microscopic observation revealed the presence of protoscoleces or their hooks in 15 CE cysts, resulting in fertility, while the remaining three resulted in sterility. Finally, the remaining two cysts did not present the typical features related to echinococcal cysts.

*Histopathological analysis*. Finally, histopathology confirmed the results of the CE parasitological analyses for the 18 samples and the two neoformations that showed different etiological causes, attributable to a hepatic abscess and a neoplastic lesion, respectively (Table 3).

### 3.2. Molecular Analyses

Fifteen DNA samples (Table 4) were positive for *E. granulosus s.s.* with the routine PCR *E.g.s.s.* protocol since the amplicon of 1001 bp of the Calreticulin (cal) ngene was successfully amplified. Moreover, the two mtgenes targets, cox1 (880 bp) and nad5 (759 bp), were identified by the respective PCRs. The remaining five samples did not yield any PCR fragments since one (hCE1) was delivered to the laboratory without the inner material, two (hCE17 and hCE18) belonged to late cyst stages CE4 and CE5, and the remaining two had resulted from different etiological causes. Sanger sequencing of the PCR cox1 and nad5 products of 720 bp and 670 bp, respectively, were compared (Table 4) as displayed on phylogenetic reconstruction.

### 3.3. Phylogenetic Analyses

**Figure 1:**
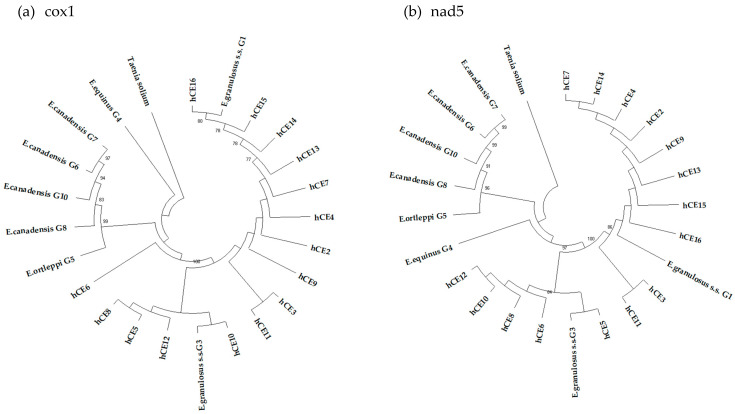
Phylogenetic trees of the 15 sequence samples collected from human patients, with reference sequences of *E. granulosus s.l.* inferred from the partial mitochondrial genes cox1 (**a**) and nad5 (**b**). *T. solium* was used as an out-group. The reliability of the tree was assessed by 1000 bootstrap replications. Bootstrap values less than 75% are not shown.

The 15 consensus sequences of partial mtgenes cox1 and nad5, isolated from each fertile echinococcal cyst (from hCE2 to hCE16) and used to build two NJ phylogenetic trees (Figure 1a,b), showed that all the samples clustered with sequences belonging to *E. granulosus s.s.*, and as inferred from the nad5 tree, 10 samples (66.7%) clustered with the G1 genotype, whereas the remaining five (33.3%) had the G3 genotype (Table 4; Figure 1).

**Figure 2:**
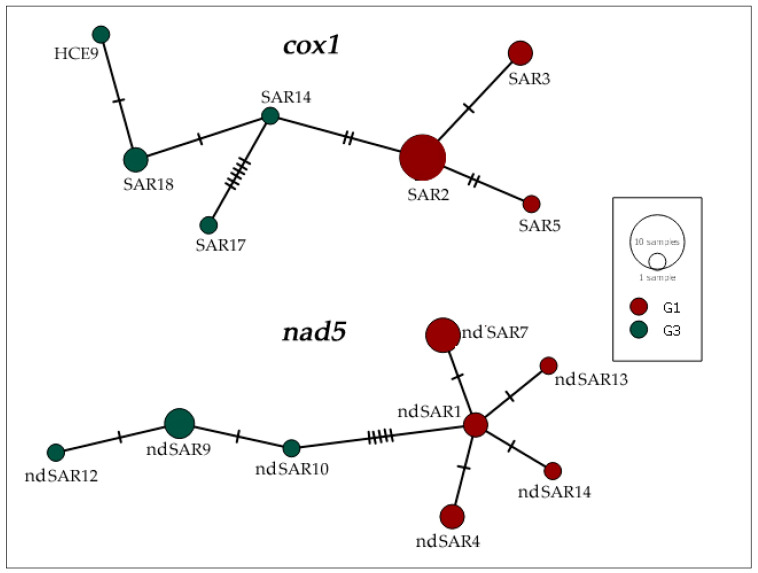
Haplotype network based on DNA sequences of the partial mitochondrial genes cox1 (720 bp) and nad5 (670 bp) of 15 *E. granulosus s.s.* isolated from human patients. Genotypes G1 (red) and G3 (green) were assigned to each haplotype based on nad5 DNA polymorphisms. Sizes of circles are proportional to the frequency of each haplotype. Numbers of mutations distinguishing the haplotypes are shown by hatch marks.

The haplotype network calculated on cox1 showed the presence of seven different haplotypes in the 15 isolates (Table 4; Figure 2). SAR2 was the most represented haplotype, since it was carried by seven cysts (hCE2, hCE4, hCE7, hCE13, hCE14, hCE15, hCE16) and SAR3 (hCE3, hCE11) and SAR18 (hCE5, hCE8) by two samples, whereas singular samples belonged to the haplotypes SAR5 (hCE9), SAR14 (hCE10), SAR17 (hCE6), and HCE9 (hCE12).

Within the nad5 sequences, a total of eight haplotypes were found (Table 4; Figure 2). The most common haplotype was nd5SAR7, grouping sequences from four subjects (hCE2, hCE4, hCE7, hCE14), whereas three samples (hCE8, hCE10, hCE12) belonged to nd5SAR9, only two (hCE15, hCE16) samples were related to nd5SAR1, and the remaining two (hCE3, hCE11) were linked to nd5SAR4. Finally, each of the remaining four haplotypes were respectively constituted by one DNA sequence: nd5SAR10 (hCE5), nd5SAR12 (hCE6), nd5SAR13 (hCE9), and nd5SAR14 (hCE13). The genotypes of *E. granulosus s.s.* samples analysed in this study were assigned on the basis of three consistently diagnostic positions of the nad5 gene fragment, as already reported [52].

## 4. Discussion

Our investigation aimed to provide a first overview of the genetic characterization of *E. granulosus s.s.* isolates from human patients. The study involved 20 subjects with suspected CE who, from 2017 to 2022, were referred to different hospitals in Sardinia (Italy) to receive emergency surgery for cyst removal: sixteen native and four from different countries. All patients were living in Sardinia (Italy) at the time of diagnosis, but four subjects were from different countries: China (hCE6), Romania (hCE13), Ghana (hCE15), and Morocco (hCE18). Since additional epidemiological data were missing, it was not possible to determine from where the infection had come. A multidisciplinary approach was used for each patient to draw a clearer clinical picture combining the clinical examination, diagnostic imaging, and laboratory analyses. The involvement of different techniques is often a fundamental approach and plays key role in ensuring the correct diagnosis of this zoonosis in human patients.

Only 15 subjects (from hCE2 to hCE15 and hCE18) of the 20 investigated presented a consistent diagnostic picture since all analyses confirmed a positive result. Consequently, these patients were undoubtedly diagnosed with CE, and the causative agent was *E. granulosus s.s.*

Conversely, various inconsistencies were found among the results obtained in a group of five patients (hCE1, hCE16, hCE17, hCE19, and hCE20). Patients hCE1, hCE16, and hCE17 had negative results by immunological tests, although the other clinical and laboratory investigations performed in this study led us to identify a typical parasitic lesion attributable to *E. granulosus*. False negative serological results have been already described in cases of hepatic CE (50–87%) for early (CE1) and late stages (CE4 and CE5) [41,42,53]. Conversely, US examination of patients hCE19 and hCE20 suspected the presence of CE cysts attributable to CE5 and CE3b stadium, respectively. However, all the other investigations performed in this study (serological, parasitological, histopathological, and biomolecular examinations) excluded *E. granulosus* infection and led us to suspect an inflammatory and neoplastic origin for hCE19 and hCE20, respectively [54]. Several previously performed studies have described guidelines for differential diagnosis between CE and other pathologies [14,55].

In agreement with what was extensively reported by other authors, hepatic localization of CE cysts was the predominant infection site of the patients in this study since the majority of cysts (87.5%) observed in this study were found in the liver. Our data were slighter more numerous than those reported elsewhere, equivalent to a range from 66% to 85% of cases [10,12,56]. Hence, the proportion of cysts found in the lungs in this study was lower (4.2%) compared to the results reported by other authors (from 15% to 47%) [56,57]. In addition, a very particular and interesting case, already described by a clinical point of view [45], harboured cysts in the lung and kidney, respectively.

In those subjects presenting with fertile echinococcal cysts, the genetic characterization of *E. granulosus s.s.* was performed. DNA-based molecular analyses represent a reliable tool for CE investigations [53], and they are useful for better defining the diagnostic path. The need for a correct CE diagnosis, specifically for humans, is fundamental to assure proper patient care and effective therapeutic treatment and follow-up. Moreover, molecular investigations help to create a more detailed picture of the biology and epidemiology of *E. granulosus* and the pathogenetic mechanisms involved in human CE. In detail, in our study, the partial ngene cal, along with the partial mtgenes cox1 and nad5, was successfully amplified from DNA extracted from the 15 CE cysts. The NJ phylogenetic tree, built on nad5 nucleotide sequences, allowed for identifying the genotypes of the 15 *E. granulosus s.s.* isolates and evinced a higher percentage of G1 (66.7%) with respect to the G3 genotype (33.3%), with no apparently specific correlation with age or gender. Only three patients from different countries, of the four investigated, presented fertile cysts, identified as two G1 (hCE13, hCE15) and one G3 (hCE6). Moreover, the G1 and G3 genotype proportions that we found in the present study are in accordance with data recorded worldwide in humans and animals by several authors. Hence, G1 has been recognized as the predominant genotype in different intermediate hosts with a cosmopolitan distribution since it can be found in different countries are continents, such as Brazil [58], China [59], Turkey [60], Iran [61], and Europe [62]. Moreover, the presence of genotype G1 was detected with high prevalence in Mediterranean countries [41], and in particular, the island of Sardinia in Italy is considered an hyperendemic area, as described in a recent study performed of 28 hydatid cysts collected from intermediate hosts of different animal species [63]. Nevertheless, there are few other countries, such as Pakistan [64] and India [65], in which the prevalent *E. granulosus s.s.* genotype is represented by G3. Considering its widespread geographical distribution, the G1 genotype is also responsible for the great majority of CE cases in humans [15,34]. Infections caused by the *E. granulosus s.s.* G3 genotype have indeed been less described [19,34].

It has been already described that a random mutation in a ngene, even it if undergoes recombination, do not show any differentiation into genetically distinct populations, as in the case of the ngene cal amplified in our study [26]. Consequently, it was not useful in providing any information about sequencing with respect to the other mtgenes.

By the analyses of our mtgene DNA samples, unlike the tree built on the cox1 mtgene, the marker nad5 allowed for a clear distinction between the G1 and G3 genotypes. As widely reported in several studies [29,30], an accurate differentiation between the G1 and G3 genotypes could not be performed by the sequencing analysis of the partial cox1 gene. In fact, as clearly described, the mtgene cox1 has an insufficient number of informative positions; consequently, it cannot clearly differentiate *E. granulosus s.s.* genotypes. Conversely, sequencing of nad5 would guarantee more consistent data for differentiation of G1 and G3, and the network analysis of nad5 confirmed better separation of the haplotypes attributed to the G1 and G3 genotypes [30,45].

As evidenced by the BLAST analysis, the majority of G1 and G3 genotypes obtained in our study were identical to other DNA sequences of various geographical origins (Mediterranean basin, Middle East, Africa, America, Asia) and different host species. Furthermore, our analyses of both mtgene fragments showed a significant level of genetic variation, as also illustrated by the haplotype networks calculated on cox1 and nad5 (Figure 2). We described a new haplotype called HCE9, including only one sample. Conversely, the SAR2 haplotype is the most represented in this study, including five Sardinian samples, one from Romania and another from Ghana, of the 15 analysed. The SAR2 haplotype is widely diffused in Mediterranean countries, as previously described in several intermediate hosts (ovine, caprine, bovine, porcine, etc.) [66]. The sample isolated from a Chinese patient, identified as the SAR17 haplotype, has never been described before in the Mediterranean area. Since additional epidemiological data were missing, we may only speculate that this haplotype could have Chinese origin.

The network analysis of nad5 showed that nd5SAR7 is the most represented haplotype belonging to the G1 genotype. The haplotype ndSAR9 included the majority of G3 genotype samples in this study and was identical to other DNA sequences of various geographical origins (Europe, Middle East, Asia) and different host species (ovine, caprine, bovine, porcine, etc.). Two new haplotypes not previously described, nd5SAR13, isolated from a Sardinian patient, and nd5SAR14, isolated from a Romanian patient, were also found. Since only a limited number of nad5 nucleotide sequences have been deposited in GenBank so far, a wider geographical distribution of the haplotypes described cannot be excluded. Further data may be revealed by future studies on a larger dataset.

Accurate genotyping has vast importance for screening highly zoonotic genetic variants of *E. granulosus s.s.*, either for epidemiological or geographical importance. In particular, it is necessary to investigate new variation, if they have a foreign origin or if these isolates come from insular territories and are located in a high endemic areas of the Mediterranean Sea, such as Sardinia [19,21,22]. Since different genetic variants may present dissimilar impacts on medical and veterinary health (e.g., infectivity of different intermediate hosts), knowledge of data on circulating genotypes among territories is of particular importance.

## 5. Conclusions

*E. granulosus s.s.* isolated from human cysts in this study presented high levels of genetic variation. Furthermore, a higher percentage of G1 (66.7%) with respect to the G3 genotype (33.3%) was also found. Finally, new haplotypes were described by the network analysis of cox1 and nad5 mtgenes; moreover, those more represented in this study were confirmed to be widespread and already described in Sardinia (Italy). Finally, important information about the molecular epidemiology of CE in Europe is provided by our data.

## Figures and Tables

**Table 2:** Description of PCR methods and primers used in the study.

Methods	Gene Amplified	Primer Sequence	Fragment Length
PCR *E.g.s.s.* [51]	Calreticulin (cal)	F/E.g ss 5′ CAATTTACGGTAAAGCAT 3′ R/E.g ss 5′ CCTCATCTCCACTCTCT 3′	1001 bp
PCR cox1 [52]	Cytochrome cox (cox1)	F/CO1: 5′ TTGAATTTGCCACGTTTGAATGC 3′ R/CO1: 5′ GAACCTAACGAGACATAACATAATGA 3′	880 bp
PCR nad5 [30]	Nicotinammide Adenin Dinucleotide 5 (nad5)	Egnd5F1: 5′ GTTGTTGAAGTTGATTGTTTTGTTTG 3′ Egnd5R1: 5′ GGAACACCGGACAAACCAAGAA 3′	759 bp

**Table 3:** Findings on imaging techniques, serology, parasitology and histopathology of the 20 patients.

Cyst/ Patients	Ultrasound Stadiation	Serological Findings	Cyst Analysis	
ID	Cyst Stadium	ELISA(OD)/IB	Parasitology	Histopathology
hCE1	CE1	negative (0.0)	positive	positive
hCE2	CE2	positive (2.6)	positive	positive
hCE3	CE2	positive (1.9)	positive	positive
hCE4	CE2	positive (1.3)	positive	positive
hCE5	CE2	positive (1.8)	positive	positive
hCE6	CE3b	positive (3.6)	positive	positive
hCE7	CE3b	positive (1.1)	positive	positive
hCE8	CE3b	positive (0.9)	positive	positive
hCE9	CE3b	positive (1.2)	positive	positive
hCE10	CE3b	positive (2.3)	positive	positive
hCE11	CE3b	positive (0.7)	positive	positive
hCE12	CE3b	positive (1.2)	positive	positive
hCE13	CE3b	positive (2.4)	positive	positive
hCE14	CE3b	positive (0.5)	positive	positive
hCE15	ND ^1^	positive (0.5)	positive	positive
	ND ^1^			
hCE16	CE3b	negative (0.0)	positive	positive
hCE17	CE4	negative (0.0)	negative	positive
hCE18	CE5	positive (2.5)	positive	positive
hCE19	CE5	negative (0.0)	negative	negative
hCE20	CE3b	negative (0.0)	negative	negative

^1^ ND: not determined.

**Table 4:** Molecular biology findings for genotype and haplotype identification.

Cyst ID/Patient	Genomic DNA Concentration	PCR cal (1001 bp)	PCR cox1 (880 bp)		PCR nad5 (759 bp)		Genotype
		Results	Sequence	Haplotype	Sequence	Haplotype	
^1^ hCE1	/	negative	/	/	/	/	/
hCE2	20.1 ng/µL	positive	MK780827	SAR2	MT993968	nd5SAR7	G1
hCE3	27.3 ng/µL	positive	MK780828	SAR3	MT993965	nd5SAR4	G1
hCE4	2.1 ng/µL	positive	MK780827	SAR2	MT993968	nd5SAR7	G1
hCE5	7.6 ng/µL	positive	MK780843	SAR18	MT993971	nd5SAR10	G3
hCE6	100.1 ng/µL	positive	MK780842	SAR17	MT993973	nd5SAR12	G3
hCE7	45.8 ng/µL	positive	MK780827	SAR2	MT993968	nd5SAR7	G1
hCE8	9.3 ng/µL	positive	MK780843	SAR18	MT993970	nd5SAR9	G3
hCE9	9.7 ng/µL	positive	MK780830	SAR5	MW287329	nd5SAR13	G1
hCE10	7.9 ng/µL	positive	MK780839	SAR14	MT993970	nd5SAR9	G3
hCE11	7.4 ng/µL	positive	MK780828	SAR3	MT993965	nd5SAR4	G1
hCE12	73.3 ng/µL	positive	MT991983	HCE9	MT993970	nd5SAR9	G3
hCE13	345.5 ng/µL	positive	MK780827	SAR2	MW287330	nd5SAR14	G1
hCE14	9.0 ng/µL	positive	MK780827	SAR2	MT993968	nd5SAR7	G1
hCE15	35.4 ng/µL ^2^ 13.0 ng/µL ^3^	positive	MK780827	SAR2	MT993962	nd5SAR1	G1
hCE16	65.70 ng/µL	positive	MK780827	SAR2	MT993962	nd5SAR1	G1
hCE17	/	negative	/	/	/	/	/
hCE18	/	negative	/	/	/	/	/
hCE19	/	negative	/	/	/	/	/
hCE20	/	negative	/	/	/	/	/

^1^ Devoid of content; ^2^ kidney; ^3^ lung.

## Data Availability

The dataset produced in this study is openly available in GenBank, see Table 4 for accession number. Moreover, authors are available to provide all the necessary information and data.
